# Niche Separation of Ammonia Oxidizers in Mudflat and Agricultural Soils Along the Yangtze River, China

**DOI:** 10.3389/fmicb.2018.03122

**Published:** 2018-12-18

**Authors:** Xue Zhou, Bolun Li, Zhiying Guo, Zhiyuan Wang, Jian Luo, Chunhui Lu

**Affiliations:** ^1^State Key Laboratory of Hydrology-Water Resources and Hydraulic Engineering, Hohai University, Nanjing, China; ^2^School of Geographic Sciences, Nanjing University of Information Science and Technology, Nanjing, China; ^3^State Key Laboratory of Soil and Sustainable Agriculture, Institute of Soil Science, Chinese Academy of Sciences, Nanjing, China; ^4^Center for Eco-Environmental Research, Nanjing Hydraulic Research Institute, Nanjing, China; ^5^School of Civil and Environmental Engineering, Georgia Institute of Technology, Atlanta, GA, United States

**Keywords:** agricultural soil, mudflat, ammonia-oxidizing archaea, ammonia-oxidizing bacteria, agricultural practice, MNTD

## Abstract

Nitrification driven by ammonia oxidizers is a key step of nitrogen removal in estuarine environments. Spatial distribution characteristics of ammonia-oxidizers have been well understood in mudflats, but less studied in the agricultural soils next to mudflats, which also play an important role in nitrogen cycling of the estuarine ecosystem. In the present research, we investigated ammonia oxidizers’ distributions along the Yangtze River estuary in Jiangsu Province, China, sampling soils right next to the estuary (mudflats) and the agricultural soils 100 m away. We determined the relationship between the abundance of *amoA* genes of ammonia-oxidizing archaea (AOA) and ammonia-oxidizing bacteria (AOB) and the potential nitrification rates of the mudflats and agricultural soils. We also identified the environmental variables that correlated with the composition of the ammonia oxidizers’ communities by 16S rRNA gene pyrosequencing. Results indicated that agricultural soils have significantly higher potential nitrification rates as well as the AOA abundance, and resulted in strong phylogenetic clustering only in AOA communities. The ammonia oxidizers’ community compositions differed dramatically among the mudflat and agricultural sites, and stochasticity played a dominant role. The AOA communities were dominated by the Group 1.1a cluster at the mudflat, whereas the 54D9 and 29i4 clusters were dominant in agriculture soils. The dominant AOB communities in the mudflat were closely related to the *Nitrosospira* lineage, whereas the agricultural soils were dominated by the *Nitrosomonas* lineage. Soil organic matter and salinity were correlated with the ammonia oxidizers’ community compositions.

## Introduction

Estuaries are mixing zones of coastal seawater and freshwater from continental runoff, acting as a conduit for the transport, transformation and production of organic carbon and nutrients. Nitrogen pollutants, emitted from both industrial and agricultural activities such as wastewater treatment plants, chemical and manufacturing industries, and livestock farming (Chung et al., 1996; Busca and Pistarino, 2003; Kim et al., 2007), are transferred from terrestrial ecosystems to aquatic ecosystems through mudflats by gravitational movement (Likens and Bormann, 1974). Coupled nitrification-denitrification can remove a substantial percentage (10∼80%) of anthropogenic nitrogen pollution in estuary ecosystems (Seitzinger, 2008), where nitrification converts ammonia to nitrite and nitrate, which are subsequently released to the atmosphere as N_2_ gas via denitrification or anammox. The removal of nitrogen pollutants in estuaries is a microbially mediated process. Because different nitrogen-cycling microbes own different activities, the niche separation of nitrogen-cycling microbes in estuaries are thus of great concern (Bernhard et al., 2007, 2010; Mosier and Francis, 2008; Moin et al., 2009; Jin et al., 2011; Damashek et al., 2015; Damashek and Francis, 2017; Zhang et al., 2018). In addition, increasing human disturbance in mudflats has a significant impact on changing soil properties, such as soil carbon and nitrogen contents (Wang et al., 2010), which are key environmental factors controlling the community composition of microorganisms involved the in nitrogen cycle. The ammonia oxidation process is the first and rate-limiting step of nitrification. It is necessary to assess the potential nitrification rates and niche differentiation of ammonia oxidizers in both multiflats and adjacent agricultural soils.

**FIGURE 1:**
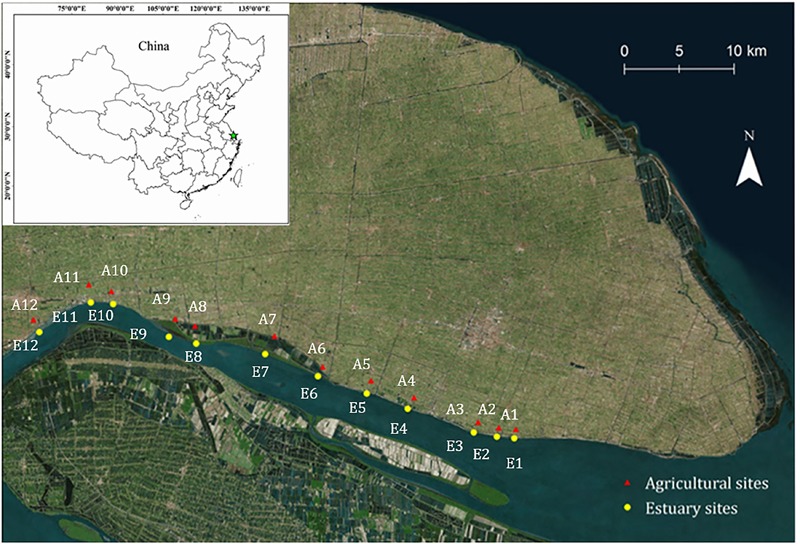
Map showing the location of sampling sites. Mudflat sites, E1–E12 (yellow circles); Agricultural sites, A1–A12 (red triangles).

The study site of the current study was the Yangtze River, the third-largest river in the world, which has a high biogeochemical flux of nitrogen, discharging into the East China Sea with a nitrate load of 6.3 × 10^6^ t/a. Intensive agricultural practice occurs in the upper estuary in Jiangsu Province, as shown in Figure 1. Ammonia oxidation is considered to be carried out in soils by ammonia oxidizing bacteria (AOB) (Kowalchuk and Stephen, 2001) and ammonia oxidizing archaea (AOA) (Leininger et al., 2006). Previous studies in open ocean environments indicated that AOA far outnumber AOB (Wuchter et al., 2006; Mincer et al., 2007; Beman et al., 2008), suggesting a high tolerance of AOA to salinity pressures (Santoro et al., 2008; Bernhard et al., 2010). However, Zhang et al. (2018) showed the equal numbers of AOA and AOB *amoA* genes at the soil/sediment interface, while AOB was more abundant than AOA in high salinity environments (Santoro et al., 2008). In addition, debatable results were also established in agricultural ecosystems. Many studies indicated the influence of ammonia concentration and fertilization on the niche separation of AOA and AOB, that AOB rather than AOA grew in soils treated with high levels of inorganic ammonium (Di et al., 2009, 2010; Jia and Conrad, 2009). In contrast, evidence from stable isotope probing (SIP) in paddy soils demonstrated that active AOA numerically outcompeted AOB (Wang et al., 2014). Thus, questions remain concerning their relative importance in mudflat and agricultural soils.

The objectives of this study were to elucidate the differentiation of the potential nitrification rates and niche separation of community composition of ammonia oxidizers (AOA and AOB) between mudflat and adjacent agricultural soils and to investigate the potential links between the environmental variables and ammonia oxidizers’ community structure.

## Materials and Methods

### Sampling

Two geographic sampling regions were selected in our study: the mudflat sites (sites E1–E12) and agricultural sites (sites A1–A12) (Figure 1). The main crop is rapeseed, which is the world’s second most important oilseed crop. Soil samples were collected in April 2017 to a depth of 0–5 cm. At each sampling location, 3 replicates of soil samples were collected. Each replicate was arranged into two randomized blocks and then mixed. All the sampled soils were sieved (<2 mm) to remove aboveground plant materials, roots, and stones. The fresh soil samples were stored in -20°C for the analysis of nitrification potential and ammonia oxidizers’ community, while dried and ground samples were used for the analysis of soil chemical properties.

Soil properties were determined as previously described (Zhou et al., 2015). Soil pH and salinity were determined in soil samples of a 1:2.5 (weight: volume) ratio with distilled water using pH and conductivity probes (Jiang et al., 2007). Soil organic matter was determined by the Walkley–Black acid digestion method, which uses chromic acid to measure the oxidizable organic carbon in the soil (Walkley and Black, 1934). NH_4_^+^-N and NO_3_^-^-N were extracted with 2M KCl at a soil/solution ratio of 1:5 by shaking at 200 rpm for 60 min and determined by a Continuous Flow Analyzer (San++System, Skalar, Holland). Soil chemistry measures normalized to dry or wet soil weight.

### Nitrification Rates

Potential nitrification rates were measured in triplicates by the shaken-slurry method as described previously (Yao et al., 2011). Ten grams of soil from each sample were preincubated at room temperature for 7 days and then mixed with 100 ml of 1.5 mM ammonium sulfate. After incubation for 0, 3, and 7 days, 10-ml slurry samples were centrifuged and the supernatant was filtered through a 0.45-μm-pore-size membrane. NO_3_^-^-N content in the supernatant was immediately analyzed and, as described above for KCl-extracted NO_3_^-^-N. NO_3_-N, concentration increased linearly and nitrification potential was calculated from the rate of increase in NO_3_^-^–N concentration over time in the slurry using linear regression.

The net nitrification rates (n) were calculated by the formula presented by Persson and Wirén (1995) as follows:

n(mgNkg-1soil day-1)=(NO3--N)t2−(NO3--N)t1t

where (NO_3_^-^-N)/t2 and (NO_3_^-^-N)/t1 are the concentrations of NO_3_^-^-N in the soil at time t2 and time t1, respectively, and t is the number of days between t2 and t1.

### DNA Extraction and *amoA* Gene Abundance

DNA was extracted from 0.5 g of each homogenized sample using the FastDNA spin for Soil Kit (Qbiogene, Inc., Irvine, CA, United States). The methods followed the manufacturer’s protocol. The quality and abundance of the DNA extractions were measured by gel electrophoresis (0.8% agarose) and NanoDrop spectrophotometer (NanoDrop Technologies Inc., Wilmington, DE, United States), and stored at -80°C.

Functional marker genes encoding *amoA* of AOA and AOB were quantified by real-time PCR (q-PCR) using the primer pairs of Arch-amoAF/Arch-amoAR (Francis et al., 2005) and amoA-1F/amoA-2R (Rotthauwe et al., 1997), respectively (Supplementary Table S2). Real-time quantitative PCR was carried out on a CFX96 Optical Real-Time Detection System (Bio-Rad Laboratories, Inc., Hercules, CA, United States). Quantitative PCR reactions were performed in 20-μL reaction mixtures containing 10 μL 2x SYBR Premix Ex Taq (Takara Biotech, Dalian, China), 200 nM of each primer, and 20 ng DNA template. The thermal program for the real-time PCR assay is shown in Supplementary Table S2. Blanks were run with water, instead of soil DNA extract, as the template to serve as the negative control. To ensure that there was no significant effect of PCR inhibition on the q-PCR data, a dilution series of templates, encompassing the working concentration, from each site were assayed using the archaeal *amoA* qPCR assay. A linear correlation was established between the dilution and copy number (*R* > 0.99), indicating no significant effect of PCR inhibitors on gene quantification. Standards for qPCR were generated using a serial dilution of known copies of PCR fragments of the respective functional gene generated using M13 PCR from clones. Standard curves, spanning 10^7^-10^1^, were constructed by a dilution series of plasmids harboring the *amoA* gene. The plasmid was extracted by the Plasmid Purification Kit (Takara) and the concentration was measured by a NanoDrop ND-1000 UV-Vis Spectrophotometer and used for the calculation of standard copy numbers. The amplification efficiency ranged from 98 to 110% with *R*^2^ values of approximately 0.99 in each reaction. Special amplification of *amoA* genes was confirmed by agarose gel electrophoresis and melting curve analysis.

### Pyrosequencing

Universal primer pair Tag-515F and 907R (Turner et al., 1999) were used to amplify soil bacterial 16S rRNA gene fragments for the MiSeq Illumina Sequencing Platform. PCR was carried out in 50-μl reaction mixtures containing each deoxynucleoside triphosphate at a concentration of 1.25 mM, 1 μl of forward and reverse primers (20 mM), 2U of Taq DNA polymerase (TaKaRa, Japan), and 50 ng of DNA. The following cycling parameters were used: 5 min of incubation at 94°C, followed by 32 cycles of 94°C for 30 s, 55°C for 30 s, and 72°C for 45 s, and a 10 min extension at 72°C (Supplementary Table S2). Triplicate reaction mixtures per sample were pooled together and purified using an agarose gel DNA purification kit (TaKaRa), and quantified using NanoDrop ND-1000 (Thermo Scientific, United States). The bar-coded PCR products were pooled in equimolar amounts (10 pg for each sample) before sequencing as described in Xiang et al. (2016). Sequences were merged by FLASH (Mago and Salzberg, 2011) and then processed using sequence data through the program AmpliconNoise, version 1.24, for the detection and correction of probable errors (Quince et al., 2011), obtainable from Quantitative Insights Into Microbial Ecology (QIIME^1^) (Caporaso et al., 2010). Poor-quality sequences (below an average quality score of 25) and short sequences (<200 bp) were removed (Supplementary Table S3). Sequences of each replicate were merged, and then clustered into Operational Taxonomic Units (OTUs) using a 97% identity threshold (default QIIME settings) by UCLUST (Edgar, 2010) and all singleton OTUs were deleted. The ammonia oxidizers’ sequences were screened at the phylum level for Thaumarchaea and at the genus levels for both AOB (*Nitrosospira* and *Nitrosomonas*), and the representative sequences were extracted for phylogenetic analysis (Supplementary Figures S3, S4).

### Phylogenetic Analysis and Data Analysis

Phylogenetic analysis of the Thaumarchaea 16S rRNA gene sequenced in this study was performed using the Molecular Evolutionary Genetics Analysis (MEGA 4.0) software package (Kumar et al., 2004). The basic tree of sequences from known AOA and AOB cultures was constructed through a neighbor-joining algorithm. The tree topology was checked using the neighbor-joining algorithm and the minimum evolution method. The nucleotide sequences of the representative OTUs were deposited in the NCBI (National Center for Biotechnology Information) under the accession numbers MH115263 to MH115276 (AOA) and MH115277 to MH115284 (AOB) of the 16S rRNA genes, respectively. The pyrosequencing reads of the total 16S rRNA genes were deposited in the NCBI under accession number SRP136463.

### Microbial Community Characterization

Sequences with 97% similarity were clustered into operational taxonomic units (OTUs) before matching the resulting consensus sequence of each OTU to known sequences in the SILVA database. Each OTU was then used as a proxy for representing a microbial species present in a sample, where the relative abundance of an OTU was directly proportional to the abundance of the microbial taxa that this OTU describes.

### Statistical Analysis

Non-metric multidimensional scaling analyses (NMDS) were performed with Bray-Curtis dissimilarity for the microbial community data. Canonical Correspondence Analysis (CCA) and BIO-ENV were used to identify the relationship between the ammonia oxidizers’ community structure and environmental parameters. The NMDS, CCA and BIO-ENV analysis were conducted using the “vegan” R package^2^.

In order to evaluate the phylogenetic community composition within each sample, the net relatedness index (NRI) was calculated by the mean phylogenetic distance (MPDs) among all co-occurring individuals, indicating the ‘basal’ dispersion of the lineages within the community. The nearest taxon index (NTI) measured the mean nearest taxon distance (MNTD) among individuals, and estimated the ‘terminal’ phylogenetic dispersion of the community (Webb, 2000). For a single community, NRI or NTI greater than +2 or less than -2 indicated that coexisting taxa were more or less related than expected by chance (phylogenetic clustering), respectively. A positive mean NRI and NTI of all communities suggested that communities were more affected by phylogenetically clustering than dispersal limitation, and a negative mean implied that communities were phylogenetically over-dispersed. NRI and NTI were calculated in the R “picante” package (Purcell et al., 2007). Beta nearest taxon index (betaNTI) was the number of standard deviations of the observed betaMNTD from the mean of null distribution (Stegen et al., 2012). If betaNTI values were below -2 or above +2, deterministic processes were dominant in shaping the community composition across all sites, and if betaNTI values were between -2 and 2, stochastic processes played an important role. The betaNTI was calculated in phylocom4.2 (Hardy, 2008). The potential relationship between *amoA* gene abundance and 16S rRNA relative abundance of AOA and AOB and environmental characteristics was analyzed by SPSS version 10.0 (IBM Co., Armonk, NY, United States), and multicollinearity was ruled out. Differences at *P* < 0.05 were considered statistically significant.

## Results

### Soil Properties

**FIGURE 2:**
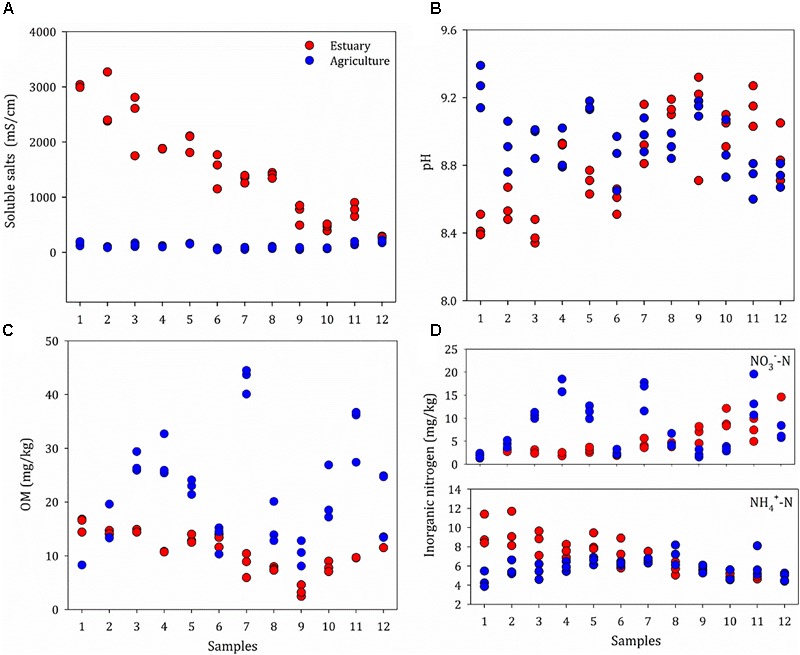
Properties of soils at the mudflat (Estuary) and agricultural (Agriculture) sites, including Soluble salts **(A)**, pH **(B)**, OM **(C)** and Inorganic nitrogen **(D)**. Original values are given in Supplementary Table S1. OM, organic matter.

The basic physio-chemical parameters of the sites are given in Figure 2 and Supplementary Table S1. The sites were defined as mudflat (prefix E) or agriculture (prefix A); the agricultural sites are about 100 meters away from the mudflat. A salinity gradient from 243 to 3000 μS/cm was observed from the upper estuary (E12) to the lower estuary (E1). The salinity ranged from 46.2 to 226 μS cm^-1^ in the agriculture sites and no spatial heterogeneity was found. A similar distribution pattern was observed for the concentration of NH_4_^+^, which increased from the upper estuary (4.2 mg kg^-1^) to the lower estuary (11.4 mg kg^-1^), with no significant difference between the agriculture sites (*P* > 0.05). The salinity was positively correlated with the concentration of NH_4_^+^ (*P* < 0.05). The organic matter content ranged from 8.08 to 16.8% along the estuarine sites (mean 11.5%), whereas the mean organic matter content for the agricultural sites was 21.0%. There was no significant difference in soil pH between the mudflat (pH 8.3–9.3) and agricultural (pH 7.9–9.4) sites.

### Potential Rates of Nitrification

**FIGURE 3:**
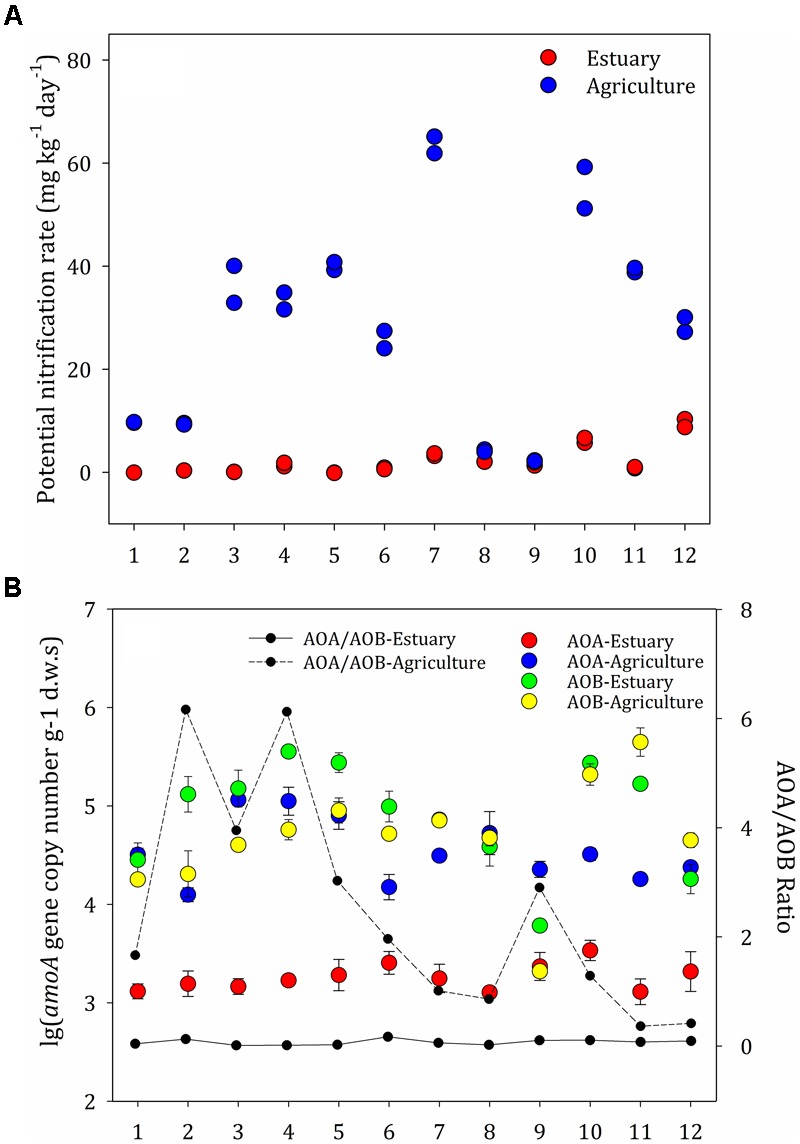
**(A)** Potential nitrification rates at the mudflat and agricultural sites. **(B)** Abundance of ammonia-oxidizing archaea (AOA) and ammonia-oxidizing bacteria (AOB) along the mudflat and at the agricultural sites based on the amoA copy numbers per gram dry weight of soil. The ratios of AOA to AOB amoA gene copies are shown as black (mudflat samples) and dotted (agricultural samples) lines. The error bars indicate the standard deviation.

The rate of nitrification at the agricultural sites ranged from 2.0 to 65.1 μg g^-1^ day^-1^ and was generally higher than the rates at the mudflat sites (0–10.3 μg g^-1^ day^-1^) (Figure 3A). The soil nitrification rates at the upper estuary sites (E7–E12) were significantly higher than those in the lower estuary sites (E1–E6). Strong positive correlations between the amount of soil organic matter and the potential nitrification rates were observed among the agricultural soils (*P* < 0.05), indicating that organic matter was the key factor influencing the potential nitrification rate in agricultural sites. Contrarily, no correlation was observed between environmental factors and the rates of nitrification at the mudflat sites.

### Abundance of Ammonia Oxidizers

The distribution pattern for the ammonia oxidizers was significantly different between the mudflat and agricultural soils (Figure 3B). Across all mudflat sites, the ammonia oxidizers’ communities were dominated by AOB (inferred from the *amoA* genes from AOB, ranging from 1.3 × 10^4^ to 1.2 × 10^5^ copies g^-1^ d.w.s), which were significantly more abundant than AOA (1.3 × 10^3^–3.4 × 10^3^ copies g^-1^ d.w.s). At the majority of the agricultural sites (A1, A2, A3, A4, A5, A6, A7, A9, and A10), the abundance of archaeal *amoA* genes (6.1 × 10^3^–3.6 × 10^5^ copies g^-1^ d.w.s) was significantly higher than the abundance of bacterial *amoA* genes (2.1 × 10^3^–2.1 × 10^5^ copies g^-1^ d.w.s) (*P* < 0.05), whereas the abundance of bacterial *amoA* genes was higher than the abundance of archaeal *amoA* genes at the other agricultural sites. The AOA:AOB ratios were between 0.01 and 0.17 at the mudflat sites and between 0.36 and 6.17 at the agricultural sites. There was no correlation between the abundance of archaea organisms and the potential nitrification rates.

### Community Structure of Ammonia Oxidizers

**FIGURE 4:**
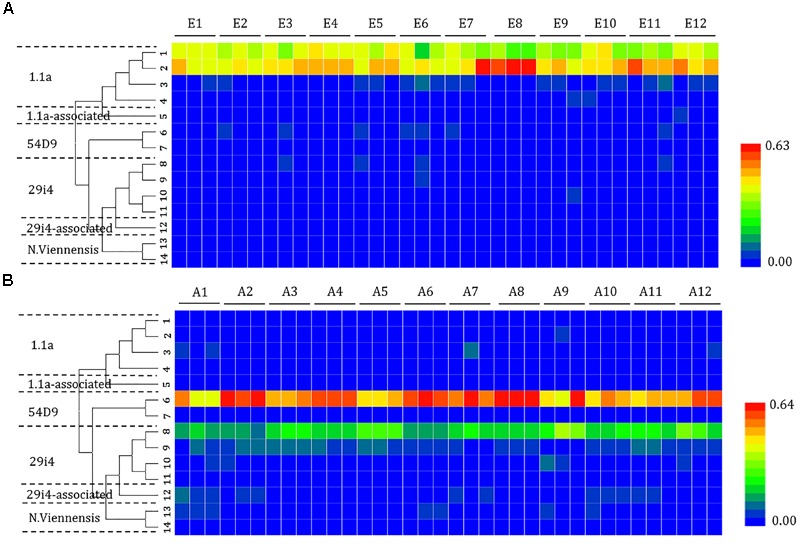
Heat map showing the relatedness of the ammonia-oxidizing archaea (AOA) community structures in the analysis of the **(A)** mudflat and **(B)** agricultural sites based on the relative abundance of distinct lineages of ammonia-oxidizing archaea 16S rRNA genes analyzed using pyrosequencing. The relative abundance of each lineage in each soil is displayed as a heat map representation. The phylogenetic position of each representative operational taxonomic unit is shown in the phylogenetic tree on the left-hand side.

**FIGURE 5:**
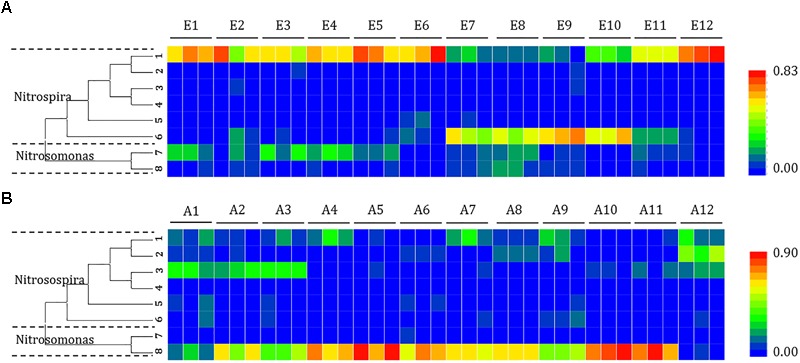
Heat map showing the relatedness of ammonia-oxidizing bacteria community structures in the analysis of the **(A)** mudflat and **(B)** agricultural sites based on the relative abundance of distinct lineages of ammonia-oxidizing bacteria 16S rRNA genes analyzed using pyrosequencing. The relative abundance of each lineage in each soil is displayed as a heat map representation. The phylogenetic position of each representative operational taxonomic unit is shown in the phylogenetic tree on the left-hand side.

The composition of the phylotypes of the ammonia oxidizers was determined by pyrosequencing samples taken from each of the sites. The 16S rRNA sequences of the ammonia oxidizers showed the presence of AOA (14 OTUs) (Figure 4) and AOB (8 OTUs) (Figure 5). The AOA OTUs could be classified into six clusters: four (OTUs 1, 2, 3, and 4) were related to the Group 1.1a cluster; one (OTU 5) was related to the Group 1.1a-associated cluster; and nine OTUs were related to the Group 1.1b cluster, including two (OTUs 6 and 7) related to the 54D9 cluster, four (OTUs 7, 8, 9, 10, and 11) related to the 29i4 cluster, one (OTU 12) falling within the 29i4-related cluster and two (OTUs 13 and 14) that most likely matched the *Nitrososphaera viennensis* cluster. The majority of AOA communities in the mudflat sites were in Group 1.1a (including OTUs 1 and 2), a cluster well-known in marine environments, with relative abundances ranging from 67.7 to 98.4%. In contrast, the dominant AOA phylotype in agricultural soils fell within clusters 54D9 (OTU 6) and 29i4 (OTU 8), with abundances ranging from 42.8 to 68.4% and 10.5 to 39.6%, respectively.

Changes in the distribution of phylotypes were also detected in the AOB communities. All the sequences of the AOB matched those of the well-described AOB in the *Nitrosospira* (OTUs 1, 2, 3, 4, 5, and 6) and *Nitrosomonas* (OTUs 7 and 8) lineages. A significant portion of the OTUs (OTU 1 at sites E1, E2, E3, E4, E5, E6, E11, and E12; OTU 6 at sites E7, E8, E9, and E10) detected at the mudflat sites belonged to the AOB *Nitrosospira* (ranging from 41.9 to 88.4% and 42.7 to 72.9%, respectively), whereas the AOB *Nitrosomonas* OTU 8 was dominant at the agricultural sites (ranging from 42.9 to 96.7%), except at site A1 (OTU 3) and A12 (OTU 2), where *Nitrosospira* was the major lineage.

**FIGURE 6:**
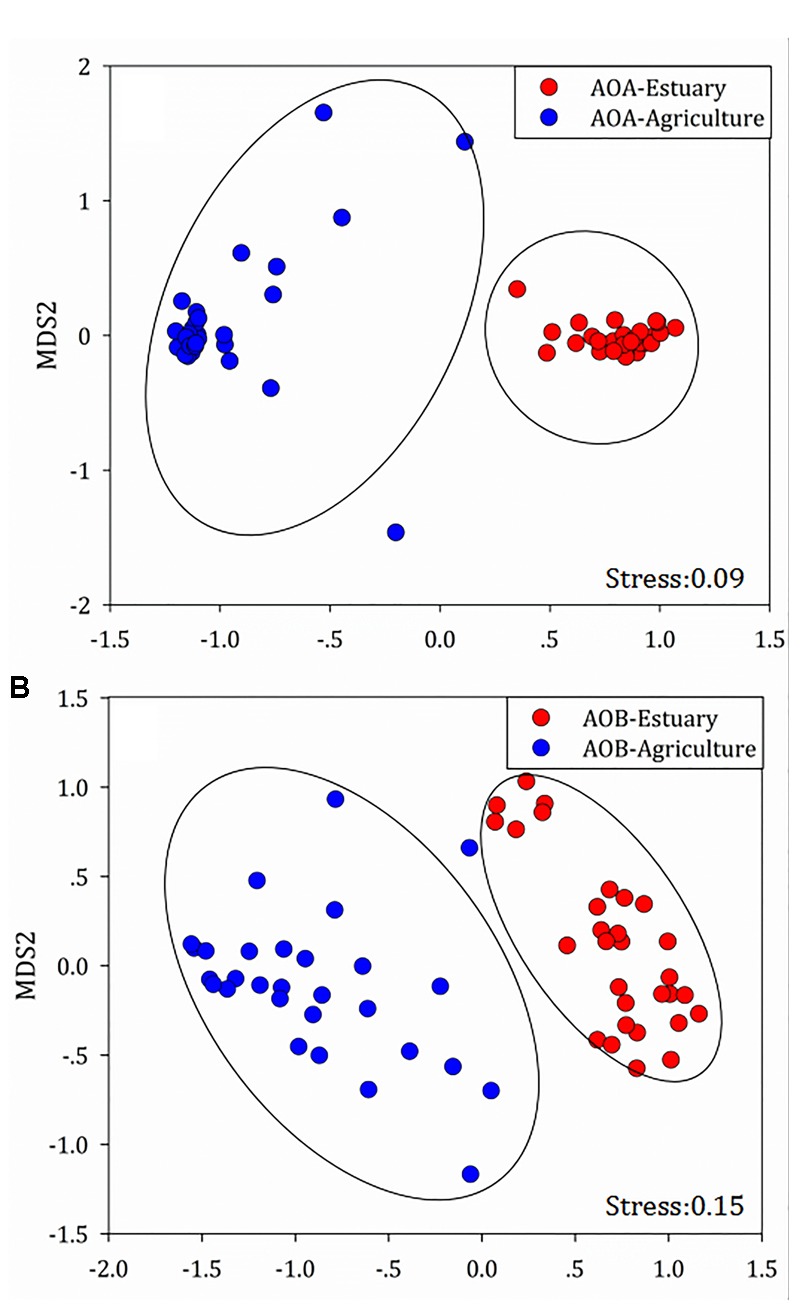
Compositional structure of **(A)** ammonia-oxidizing archaea (AOA) and **(B)** ammonia-oxidizing bacteria (AOB) 16S rRNA genes among the mudflat and agricultural sites as described by non-metric multidimensional scaling plots.

Non-metric multidimensional scaling (NMDS) analysis was used to demonstrate the structural divergence of the community composition and to show the community differentiation of AOA and AOB between the mudflat sites and the agricultural sites (Figure 6).

### Contribution of Deterministic and Stochastic Processes Control Ammonia Oxidizers’ Community Composition

**FIGURE 7:**
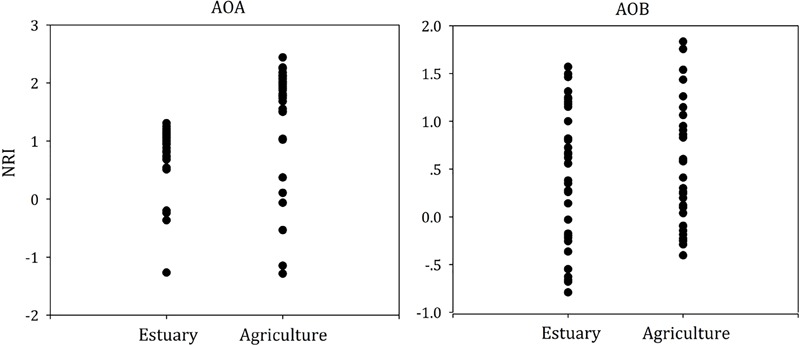
Phylogenetic diversity metrics (NRI) for AOA and AOB community assemblages.

To test whether dispersal limited or niche-based mechanisms better explained the assembly of ammonia oxidizers’ community composition in mudflat and agricultural sites, we calculated the NRI (Figure 7) and NTI (Supplementary Figure S1) for each single community.

For the AOA and AOB, we found that the mean NRI and mean NTI were positive (Figure 7 and Supplementary Figure S1, *P < 0.05*), suggesting AOA and AOB communities in both mudflat and agricultural soils were more influenced by niche processes than dispersal limitation. For the AOA, the mean value of NRI in agricultural soils was higher than that in mudflat sites, whereas no significant difference of the mean NTI value was observed between mudflat and agricultural soils. In addition, the mean NRI value of AOA was above 2 in agricultural soils. For AOB, no differentiation of either the mean NRI or NTI value was observed between mudflat and agricultural soils, and the NRI and NTI scores in all samples were between -2 and 2.

In order to establish which process controlled community dynamics at different spatial scales, βNTI values were determined. The results showed that a stochastic process was dominant at all the sites in our study (Supplementary Figure S2; value > -2 and <2).

### Relationship Between Environmental Parameters and the Community Structure of Ammonia Oxidizers

**FIGURE 8:**
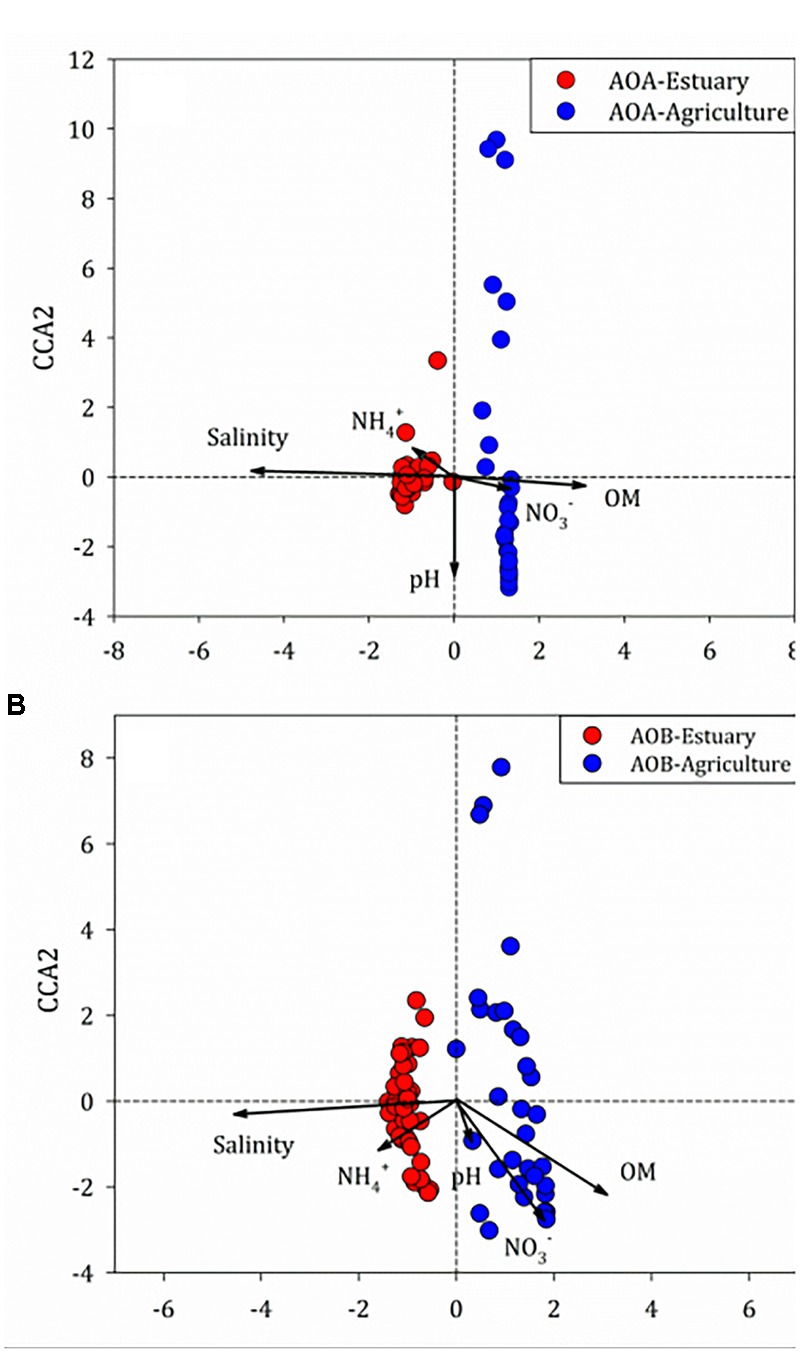
Canonical correspondence analysis of environmental variables and **(A)** ammonia-oxidizing archaea (AOA) and **(B)** ammonia-oxidizing bacteria (AOB) 16S rRNA genes among the mudflat and agricultural sites.

There was no correlation between the abundance of *amoA* genes and environmental parameters at any of the mudflat or agricultural soils. Potential relationships between the composition of the ammonia oxidizers’ community and the soil physico-chemical properties were inferred through unconstrained (BIO-ENV) (Clarke and Ainsworth, 1993) and constrained (CCA) multivariate methods based on the relative abundance of the OTUs of the ammonia oxidizers (Figure 8). The BIO-ENV analysis showed that soil salinity had the best individual associations with the composition of AOA, whereas soil organic matter explained the composition of AOB (Supplementary Tables S4, S5). Two axes of the CCA explained 38.6% of the total variance in the AOA 16S rRNA genotype composition and 73.9% of the cumulative variance of the genotype–environment relationship. Only soil salinity and organic matter significantly influenced the distribution of the AOA 16S rRNA genotypes (*r*^2^ = 0.52, *P* < 0.001; *r*^2^ = 0.23, *P* < 0.001, respectively, 999 permutations). For the AOB, two axes of the CCA explained 34.7% of the total variance in the AOB genotype composition and 64.8% of the cumulative variance of the genotype–environment relationship. The salinity (*r*^2^ = 0.55, *P* < 0.001, 999 permutations) and amount of organic matter (*r*^2^ = 0.24, *P* < 0.001, 999 permutations) significantly affected the 16S rRNA gene composition of AOB.

## Discussion

### The Effect of Agricultural Practice on Soil Potential Nitrification Rate

Agricultural practice increased the soil potential nitrification rates. The nitrification potential rates were considerably higher in the agricultural soils than those at the mudflat sites, except for sites E8 and E9, suggesting the higher organic matter concentration and lower salinity might both contribute to the increase of potential nitrification rates in agricultural soils. This is in agreement with publications reported previously, in which salinity and organic matter were important in controlling the rates of nitrification (Jiang et al., 2007; Zhou et al., 2015) as the nitrification rates generally decreased with the increasing salinity and external nitrogen addition stimulated nitrification (Offre et al., 2009; Gubry-Rangin et al., 2010; Zhang et al., 2010; He et al., 2012) by regulating the nitrifying community (Bernhard and Bollmann, 2010). Spatial differences in the nitrification potential rates were also observed in mudflat sites. The nitrification rates at the low-salinity sites (E7–E12) were significantly higher than those at high-salinity sites (E1–E6), further confirming the negative effects of salinity on nitrification rates.

It is important to note that the nitrification potential rates were determined with samples that were frozen at -20°C after thawing. Freezing/thawing will change the community structure of active microbes and activity potentials due to the disruption of cells. Soil freezing has been suggested to destroy microbes physically, releasing nutrients from the destroyed cells (Schimel and Clein, 1996; Sharma et al., 2006). The thaw-related flush of nutrients increases in activity and biomass of those microbes that survive such soil freeze (Skogland et al., 1988). However, the response pattern of AOA and AOB to thaw still evaded understanding. The *amoA* gene abundance significantly increased from -4°C to 5°C (Sophie et al., 2012), while quantities of *amoA* gene expression did not significantly change over the spring thaw (Németh et al., 2014). Thus, whether freezing/thawing soil accurately reflects soil nitrification potential rates still requires further research.

### Deterministic and Stochastic Factors on Ammonia Oxidizers’ Community

We observed stronger phylogenetic clustering in the AOA community of agricultural sites compared with the AOA community in mudflat sites (Figure 7). Although MNTD analysis also demonstrated the phylogenetic clustering effects in AOB communities of all the sites, no difference of the effects was observed between mudflat and agricultural sites, suggesting agricultural practice had stronger effects on the AOA community structure than the AOB.

We also found that the ammonia oxidizers’ community of both agricultural and mudflat sites were more strongly influenced by stochastic factors such as ecological drift, mutation, and random growth and deaths (Kembel, 2009; Fine and Kembel, 2011; Stegen et al., 2012) rather than by deterministic processes. These findings are consistent with publications reported previously, in which deterministic influences were more likely to dominate at large scales, and stochasticity trended to dominate at local scale (Shi et al., 2018).

### Community Structure of Ammonia Oxidizers in Mudflat and Agricultural Soils

Much of what we know about marine AOA has come from studies in open ocean environments, where AOA far outnumber AOB (Wuchter et al., 2006; Mincer et al., 2007; Beman et al., 2008), suggesting the high tolerance of AOA to salinity pressures (Santoro et al., 2008; Bernhard et al., 2010). In the mudflat, the dominant cluster was Group 1.1a and was highly related to *Nitrosopumilus maritimus* AOA, which has been isolated from marine environments (Konneke et al., 2005) and has a much higher tolerance of high salinity than other AOA. Based on the abundance of archaeal and bacterial *amoA* genes and AOA/AOB ratios, our study showed that the population size of AOA remained stable along the changing salinity gradient of mudflat sites. On the contrary, the abundance of AOB *amoA* gene were much higher in the high-salinity mudflat sites than those in low-salinity mudflat sites, which is consistent with previous results showing that AOB *amoA* gene abundances are greater and increase with salinity (Mosier and Francis, 2008; Santoro et al., 2008; Zhang et al., 2018), suggesting the potentially dominant contribution of AOB in mudflats. A recent study also established that the AOB community was first regulated by salinity (Zhang et al., 2018). In addition, the large shifts and contribution in the composition of the 16S rRNA and *amoA* clone libraries of AOB communities have been reported for the Schelde estuary in the Netherlands (De Bie et al., 2001; Bollmann and Laanbroek, 2002) and Waquoit Bay in the United States (Bernhard et al., 2005), indicating that salinity may affect the niche separation of AOB. However, no significantly distinct community structure of AOB was observed along the mudflat in this study. Sequences relating to *Nitrosospira* spp. were dominant at the mudflat sites ranging from low salinity to high salinity. Publications previously reported a selection for *Nitrosospira* spp. with increasing salinity in estuarine systems (Bernhard et al., 2007; Sahan and Muyzer, 2008). However, no statistical correlation was observed between nitrification rates and AOB population size, which might be attributed to the uncoupling between specific activity and the abundance of ammonia oxidizers.

Although agricultural practice in neutral soils has been widely reported with AOB activity, as AOB rather than AOA dominate oxidation of added ammonium (Xia et al., 2011; Prosser and Nicol, 2012), AOA outnumbered AOB *amoA* genes in the agricultural soils of this study, which might be attributed to the accumulation of organic matter rather than ammonia content. In fact, the significant growth of thaumarchaeota was also observed by root exudation (Chen et al., 2008) and mature slurry (Zhou et al., 2015), but less affected by the addition of inorganic fertilizers (Lehtovirta-Morley et al., 2011), indicating the importance of organic matter in the AOA growth. In contrast, AOB cannot grow on organic compounds (Krümmel and Harms, 1982). The dominant OTUs in the AOA guild for the agricultural sites were related to the 54D9 and 29i4 clusters, which have been observed to be the predominant AOA community in agricultural systems with high organic content (Bernhard and Bollmann, 2010; Zhou et al., 2015). The proposed mechanisms include that (1) mineralized ammonia was the major substrate source for thaumarchaeal nitrification processes (Stopnišek et al., 2010), (2) thaumarchaea had a mixotrophic lifestyle for assimilation of organic matters (Levičnik-Höfferle et al., 2011). The potentially hetetrophic pathway of AOA was also consistent with the positive correlation between OM and nitrification rates in agricultural sites.

## Conclusion

In summary, both potential nitrification rates and AOA abundance were higher in the agricultural soils than those in mudflat, indicating the importance of 29i4 and 54D9 AOA in agricultural soils, while AOB was dominant in mudflats. Stronger phylogenetic clustering was only observed in the AOA community under agricultural practices, suggesting the importance of AOA in agricultural soils. Our study also revealed that stochastic processes dominated in AOA and AOB communities in both mudflat and agricultural sites, suggesting stochastic processes played an important role at the small scale (22 km) of our study. Both organic matter and salinity contributed to the distinct community compositions of the AOA and AOB. These results expand our understanding of ammonia oxidizers’ biogeographic distribution in both mudflat and agricultural soils of estuarine ecosystems. To date, recent discovery of complete ammonia oxidizer (comammox) organisms, i.e., bacteria that completely oxidize ammonia to nitrate (Daims et al., 2016), within the genus Nitrospira has led to a dramatic shift in the current model of nitrification. The niche differentiation of comammox in the mudflat is worth being explored further. We should also notice that environmental parameters not measured in the study, such as oxygen concentration and metal ion concentration, are also likely to influence the community structure.

## Importance

Our study assessed the niche differentiation of ammonia oxidizers and potential nitrification rates in estuarine ecosystems in both mudflat and adjacent agricultural soils.

## Author Contributions

XZ and CL designed the experiments. XZ, BL, and ZG carried out the experiments and performed the analyses. XZ, CL, ZW, and JL substantially contributed to the interpretation of the results and writing of the paper.

## Conflict of Interest Statement

The authors declare that the research was conducted in the absence of any commercial or financial relationships that could be construed as a potential conflict of interest.

## Footnotes

1 http://www.qiime.org/

2 http://www.r-project.org/
