# Supplementary material for: Niche Separation of Ammonia Oxidizers in Mudflat and Agricultural Soils Along the Yangtze River, China
Source: Front Microbiol. 2018 Dec 18;9:3122. doi: 10.3389/fmicb.2018.03122 (PMC6305492; doi:10.3389/fmicb.2018.03122)

**Table S1** Environmental parameters of samples from the estuarine and agriculture sites.

|  | Mudflat | | | | | | Agriculture | | | | | | | Location |
| --- | --- | --- | --- | --- | --- | --- | --- | --- | --- | --- | --- | --- | --- | --- |
| Sample No. | No. of replicates | Electrical conductivity of soil solution (S/cm) | pH | NO_3_**^−^**-N (mg kg^−1^) | NH_4_**^+^**-N (mg kg^−1^) | Organic matter content (g kg^−1^) | Sample No. | No. of replicates | Electrical conductivity of soil solution (S/cm) | pH | NO_3_**^−^**-N (mg kg^−1^) | NH_4_**^+^**-N (mg kg^−1^) | Organic matter content (g kg^−1^) |  |
| E1 | 1 | 3000 | 8.5 | 2.2 | 8.7 | 14.4 | A1 | 1 | 172 | 9.1 | 2.2 | 4.2 | 8.3 | N 31°43' E 121°40' |
|  | 2 | 3040 | 8.4 | 1.3 | 8.4 | 16.8 |  | 2 | 117 | 9.3 | 2.4 | 5.5 | 8.3 |  |
|  | 3 | 2990 | 8.4 | 1.9 | 11.4 | 16.6 |  | 3 | 194 | 9.6 | 1.4 | 3.9 | 8.3 |  |
| E2 | 1 | 2380 | 8.5 | 3.0 | 4.1 | 14.0 | A2 | 1 | 106 | 8.8 | 4.4 | 5.2 | 19.6 | N 31°43' E 121°39' |
|  | 2 | 2400 | 8.5 | 3.3 | 9.1 | 14.7 |  | 2 | 86 | 9.1 | 3.6 | 5.4 | 13.5 |  |
|  | 3 | 3270 | 8.7 | 2.8 | 11.7 | 14.1 |  | 3 | 84 | 8.9 | 5.2 | 6.6 | 13.3 |  |
| E3 | 1 | 2810 | 8.3 | 2.5 | 8.8 | 14.4 | A3 | 1 | 103 | 9.0 | 9.9 | 4.6 | 25.9 | N 31°44' E 121°38' |
|  | 2 | 1750 | 8.4 | 3.2 | 9.6 | 14.9 |  | 2 | 171 | 8.8 | 10.6 | 6.2 | 26.3 |  |
|  | 3 | 2610 | 8.5 | 2.4 | 7.1 | 14.4 |  | 3 | 133 | 9.0 | 11.3 | 5.4 | 29.4 |  |
| E4 | 1 | 1872 | 8.9 | 1.8 | 6.9 | 10.8 | A4 | 1 | 122 | 8.8 | 18.5 | 6.5 | 32.7 | N 31°45' E 121°34' |
|  | 2 | 1885 | 8.9 | 1.8 | 8.2 | 10.8 |  | 2 | 105 | 8.8 | 15.8 | 5.4 | 25.4 |  |
|  | 3 | 1879 | 8.9 | 2.6 | 7.6 | 10.7 |  | 3 | 97 | 9.0 | 15.7 | 5.9 | 25.9 |  |
| E5 | 1 | 2110 | 8.6 | 2.6 | 7.9 | 12.8 | A5 | 1 | 160 | 9.2 | 9.9 | 6.1 | 21.4 | N 31°46' E 121°32' |
|  | 2 | 2100 | 8.8 | 3.1 | 7.8 | 14.0 |  | 2 | 166 | 9.1 | 12.7 | 6.9 | 23.0 |  |
|  | 3 | 1809 | 8.7 | 3.7 | 9.5 | 12.5 |  | 3 | 150 | 9.1 | 11.4 | 6.6 | 24.1 |  |
| E6 | 1 | 1769 | 8.5 | 1.9 | 7.2 | 14.0 | A6 | 1 | 70 | 8.9 | 2.2 | 6.5 | 10.3 | N 31°48' E 121°29' |
|  | 2 | 1584 | 8.7 | 2.3 | 8.9 | 11.6 |  | 2 | 78 | 8.7 | 2.2 | 6.1 | 15.2 |  |
|  | 3 | 1151 | 8.6 | 2.1 | 5.8 | 13.4 |  | 3 | 46 | 9.0 | 3.3 | 6.3 | 14.5 |  |
| E7 | 1 | 1356 | 8.7 | 4.0 | 7.5 | 8.9 | A7 | 1 | 92 | 8.0 | 17.8 | 6.5 | 40.1 | N 31°49' E 121°27' |
|  | 2 | 1256 | 8.9 | 5.7 | 6.3 | 10.4 |  | 2 | 51 | 8.1 | 11.6 | 6.3 | 43.7 |  |
|  | 3 | 1394 | 9.2 | 3.6 | 6.5 | 5.9 |  | 3 | 88 | 7.9 | 17.0 | 6.8 | 44.5 |  |
| E8 | 1 | 1446 | 9.1 | 4.7 | 5.1 | 8.0 | A8 | 1 | 109 | 8.9 | 4.3 | 8.2 | 13.9 | N 31°50' E 121°23' |
|  | 2 | 1407 | 9.2 | 4.3 | 6.4 | 7.6 |  | 2 | 67 | 9.0 | 4.0 | 6.1 | 12.8 |  |
|  | 3 | 1343 | 9.1 | 3.8 | 5.7 | 7.3 |  | 3 | 87 | 8.8 | 6.7 | 7.2 | 20.1 |  |
| E9 | 1 | 493 | 9.2 | 7.1 | 5.6 | 10.6 | A9 | 1 | 49 | 9.2 | 1.6 | 5.9 | 2.4 | N 31°50' E 121°22' |
|  | 2 | 779 | 9.3 | 4.6 | 5.5 | 8.1 |  | 2 | 66 | 9.2 | 2.1 | 6.1 | 4.6 |  |
|  | 3 | 850 | 8.6 | 8.2 | 5.6 | 12.8 |  | 3 | 89 | 9.1 | 3.2 | 5.3 | 3.2 |  |
| E10 | 1 | 391 | 8.9 | 12.1 | 4.8 | 9.0 | A10 | 1 | 61 | 9.1 | 3.9 | 5.6 | 17.2 | N 31°52' E 121°19' |
|  | 2 | 472 | 9.2 | 8.7 | 5.2 | 7.8 |  | 2 | 63 | 8.9 | 2.8 | 4.6 | 18.5 |  |
|  | 3 | 512 | 9.1 | 8.3 | 5.2 | 7.1 |  | 3 | 83 | 8.7 | 3.4 | 4.6 | 26.9 |  |
| E11 | 1 | 903 | 9.3 | 5.0 | 4.6 | 9.7 | A11 | 1 | 136 | 8.8 | 19.6 | 5.1 | 36.2 | N 31°53' E 121°18' |
|  | 2 | 651 | 9.0 | 9.9 | 5.2 | 9.6 |  | 2 | 152 | 8.6 | 13.1 | 8.1 | 36.7 |  |
|  | 3 | 777 | 9.2 | 7.5 | 4.9 | 9.7 |  | 3 | 199 | 8.8 | 10.7 | 5.6 | 27.4 |  |
| E12 | 1 | 243 | 9.1 | 14.6 | 4.4 | 13.5 | A12 | 1 | 174 | 8.8 | 5.8 | 5.3 | 24.9 | N 31°52' E 121°15' |
|  | 2 | 217 | 8.6 | 16.1 | 4.2 | 11.5 |  | 2 | 226 | 8.7 | 8.4 | 5.1 | 24.7 |  |
|  | 3 | 230 | 8.8 | 10.6 | 4.6 | 13.5 |  | 3 | 200 | 8.7 | 6.1 | 4.5 | 13.4 |  |

**Table S2.** Primers and conditions used in this study.

| Primer | Primer sequence (5′–3′) | | Target gene | Thermal profile | Reference |
| --- | --- | --- | --- | --- | --- |
| Arch-*amo*AF | STA ATG GTC TGG CTT AGA CG | Archaeal *amoA* gene | | 95°C, 3 min; 35×(95°C, 30 s; 55°C, 30 s; 72°C, 30 s with plate read); melt curve 65.0–95.0°C, increment 0.5°, 0:05+ plate read | (Francis et al., 2005) |
| Arch-*amo*AR | GCG GCC ATC CAT CTG TAT GT |  |  |  |  |
| *amoA*-1F | GGG GTT TCT ACT GGT GGT | | Bacterial *amoA* gene | 95°C, 3 min; 35×(95°C, 30 s; 55°C, 30 s; 72°C, 30 s with plate read); melt curve 65.0–95.0°C, increment 0.5°, 0:05+ plate read | (Rotthauwe et al., 1997) |
| *amoA*-2R | CCC CTC KGS AAA GCC TTC TTC | |  |  |  |
| 515F | GTG CCA GCM GCC GCG G | | Universal 16S rRNA genes | 94°C, 5 min; 32×(94°C, 30 s; 54°C, 30 s; 72°C, 45 s); 72°, 10 min; hold at 4°C | (Stubner, 2002) |
| 907R | CCG TCA ATT CMT TTR AGT TT | |  |  |  |

**Table S3.** Pyrosequencing summary of all the 16S rRNA genes in the samples tested.

| Site | Replicate | Read No. | Site | Replicate | Read No. |
| --- | --- | --- | --- | --- | --- |
| E1 | 1 | 270963 | A1 | 1 | 219330 |
|  | 2 | 36465 |  | 2 | 49603 |
|  | 3 | 57761 |  | 3 | 82564 |
| E2 | 1 | 266291 | A2 | 1 | 277828 |
|  | 2 | 45420 |  | 2 | 96025 |
|  | 3 | 38679 |  | 3 | 55965 |
| E3 | 1 | 55620 | A3 | 1 | 72967 |
|  | 2 | 91400 |  | 2 | 29581 |
|  | 3 | 28243 |  | 3 | 83721 |
| E4 | 1 | 78976 | A4 | 1 | 77470 |
|  | 2 | 79176 |  | 2 | 23959 |
|  | 3 | 99628 |  | 3 | 74271 |
| E5 | 1 | 35224 | A5 | 1 | 179452 |
|  | 2 | 29684 |  | 2 | 75387 |
|  | 3 | 43535 |  | 3 | 63404 |
| E6 | 1 | 37089 | A6 | 1 | 56065 |
|  | 2 | 62823 |  | 2 | 58875 |
|  | 3 | 62873 |  | 3 | 62604 |
| E7 | 1 | 38080 | A7 | 1 | 21623 |
|  | 2 | 24563 |  | 2 | 9030 |
|  | 3 | 18308 |  | 3 | 35291 |
| E8 | 1 | 65267 | A8 | 1 | 364416 |
|  | 2 | 63053 |  | 2 | 60095 |
|  | 3 | 35275 |  | 3 | 92478 |
| E9 | 1 | 62222 | A9 | 1 | 37036 |
|  | 2 | 79036 |  | 2 | 56639 |
|  | 3 | 47358 |  | 3 | 39926 |
| E10 | 1 | 19788 | A10 | 1 | 49547 |
|  | 2 | 74053 |  | 2 | 54378 |
|  | 3 | 27132 |  | 3 | 64792 |
| E11 | 1 | 63688 | A11 | 1 | 56625 |
|  | 2 | 74739 |  | 2 | 64034 |
|  | 3 | 29099 |  | 3 | 18940 |
| E12 | 1 | 18084 | A12 | 1 | 22276 |
|  | 2 | 58787 |  | 2 | 43509 |
|  | 3 | 143976 |  | 3 | 146640 |

**Table S4.** BIO-ENV analysis based on Speraman's rank correlation coefficient (ρ), showing the association between the composition of ammonia-oxidizing archaea (based on the relative abundance of the ammonia-oxidizing archaea operational taxonomic units detected) and environmental variables.

| Combined variables | Spearman's rank correlation coefficient (ρ) |
| --- | --- |
| Organic matter | 0.372 |
| Salinity+ organic matter | 0.367 |
| Salinity + NO_3_^−^ + organic matter | 0.330 |
| Salinity + pH + NO_3_^−^ + organic matter | 0.258 |

**Table S5.** BIO-ENV analysis based on Speraman's rank correlation coefficient (ρ), showing the association between the composition of ammonia-oxidizing bacteria (based on the relative abundance of the ammonia-oxidizing bacteria operational taxonomic units detected and environmental variables.

| Combined variables | Spearman's rank correlation coefficient (ρ) |
| --- | --- |
| Salinity | 0.321 |
| Salinity+ organic matter | 0.343 |
| Salinity + NO_3_^−^ + organic matter | 0.304 |
| Salinity + pH + NO_3_^−^ + organic matter | 0.269 |

Francis CA, Roberts KJ, Beman JM, Santoro AE, Oakley BB. Ubiquity and diversity of ammonia-oxidizing archaea in water columns and sediments of the ocean. Proceedings of the National Academy of Sciences of the United States of America 2005; 102: 14683-14688.

Rotthauwe JH, Witzel KP, Liesack W. The ammonia monooxygenase structural gene amoA as a functional marker: Molecular fine-scale analysis of natural ammonia-oxidizing populations. Applied and Environmental Microbiology 1997; 63: 4704-4712.

Stubner S. Enumeration of 16S rDNA of Desulfotomaculum lineage 1 in rice field soil by real-time PCR with SybrGreen (TM) detection. Journal of Microbiological Methods 2002; 50: 155-164.

**Figure S1.** Scatter plot of NTI values of AOA and AOB communities in each samples.

**
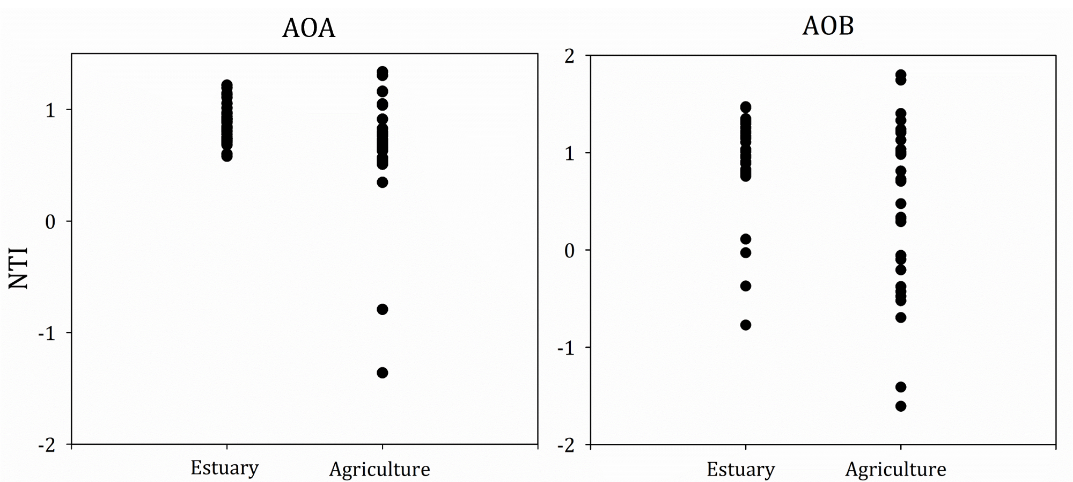
**

**Figure S2.** Scatter plot of βNTI values of AOA and AOB communities in each samples.

**

**

**Figure S3.** Phylogenetic tree of the ammonia-oxidizing archaea (AOA) 16S rRNA gene.


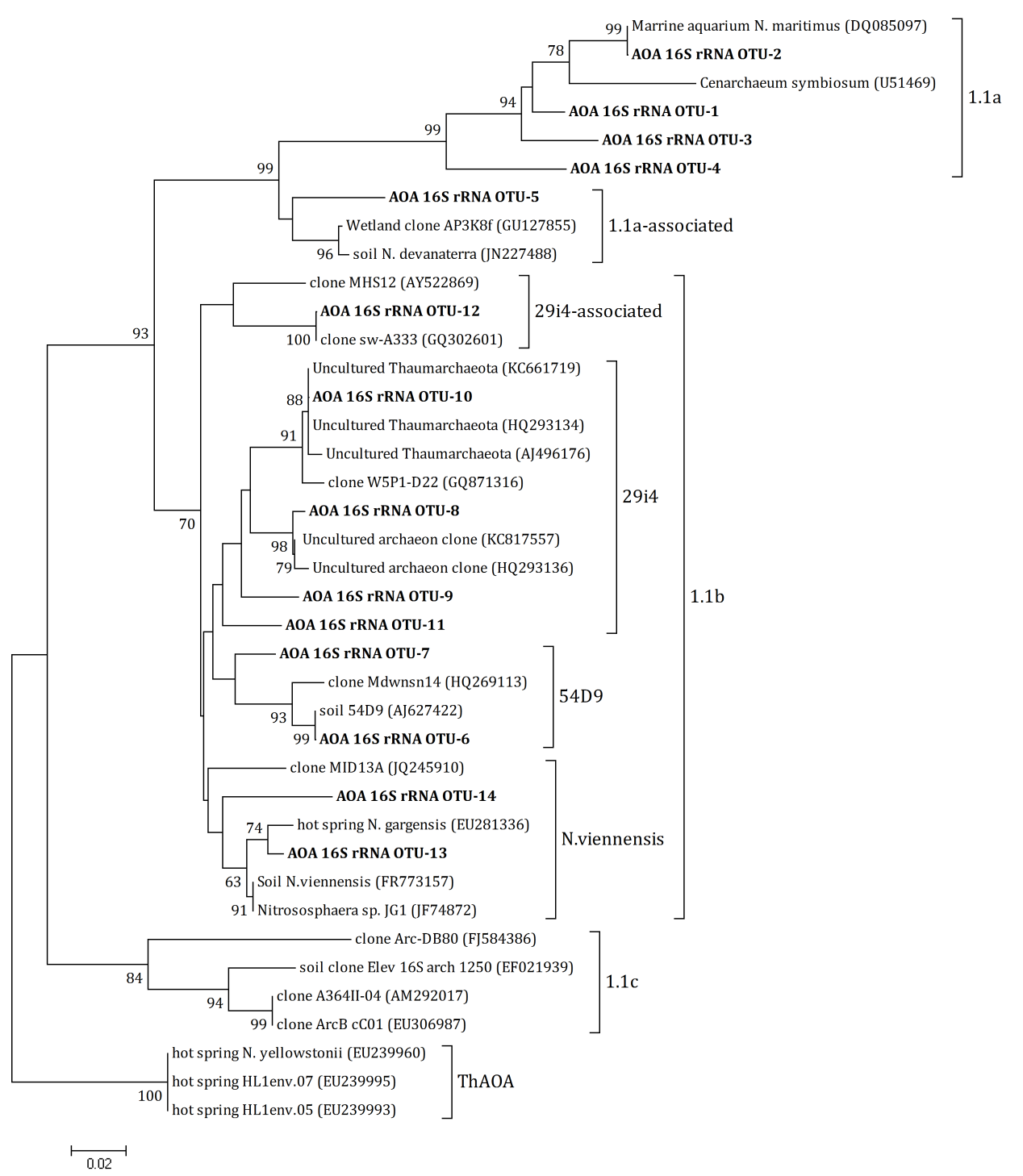


**Figure S4** Phylogenetic tree of the ammonia-oxidizing bacteria (AOB) 16S rRNA gene.


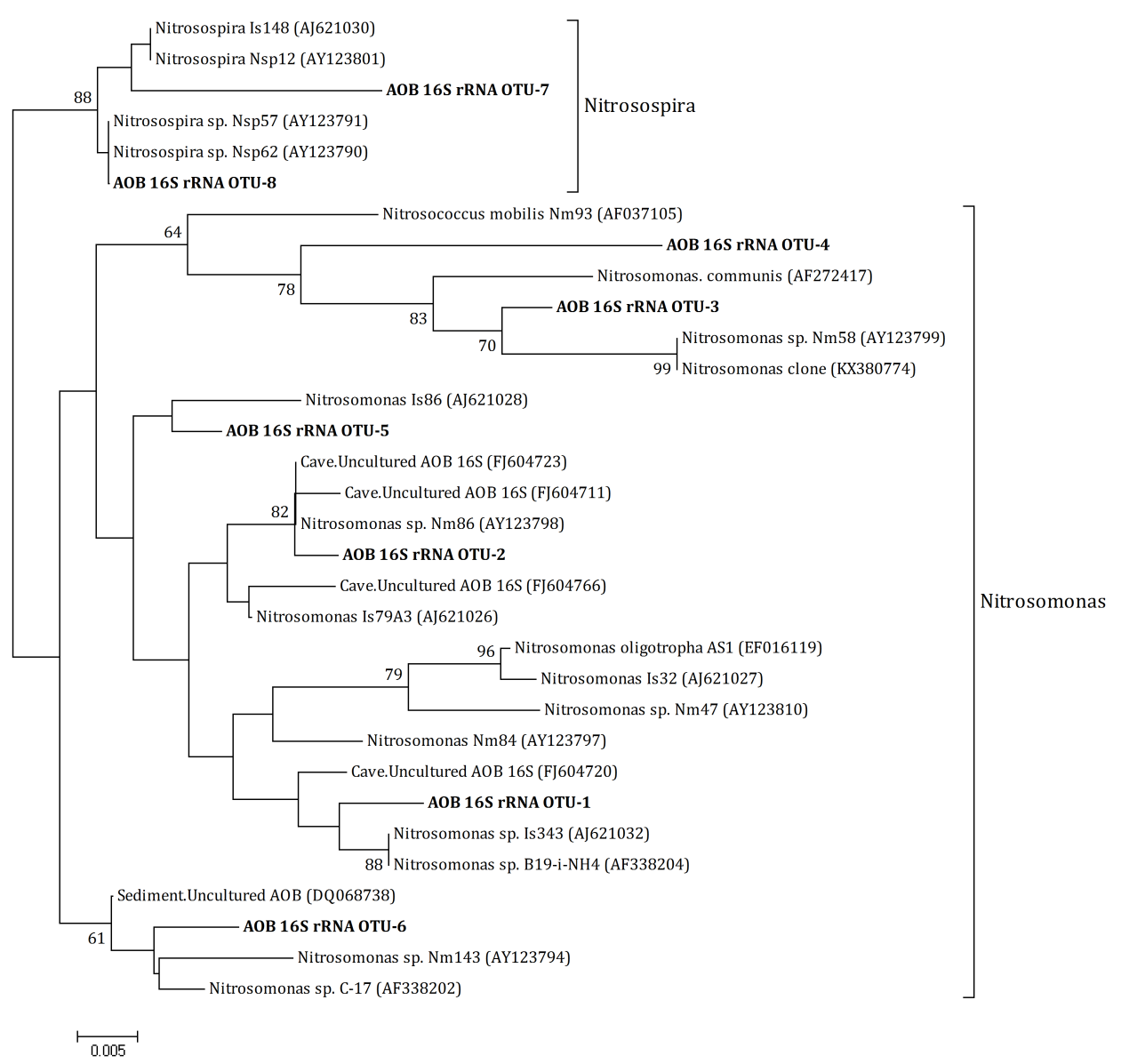

Supplement: Supplementary file 1 [file Table_1.DOCX]
